# Two-Chains Tissue Plasminogen Activator Unifies Met and NMDA Receptor Signalling to Control Neuronal Survival

**DOI:** 10.3390/ijms222413483

**Published:** 2021-12-15

**Authors:** Elodie Hedou, Sara Douceau, Arnaud Chevilley, Alexandre Varangot, Audrey M. Thiebaut, Hortense Triniac, Isabelle Bardou, Carine Ali, Mike Maillasson, Tiziana Crepaldi, Paolo Comoglio, Eloïse Lemarchand, Véronique Agin, Benoit D. Roussel, Denis Vivien

**Affiliations:** 1 Normandie University, UNICAEN, INSERM U1237, Etablissement Français du Sang, Physiopathology and Imaging of Neurological Disorders (PhIND), Cyceron, Institut Blood and Brain @ Caen-Normandie (BB@C), 14000 Caen, France; hedou@cyceron.fr (E.H.); douceau@cyceron.fr (S.D.); chevilley@cyceron.fr (A.C.); varangot@cyceron.fr (A.V.); audrey.thiebaut@sorbonne-universite.fr (A.M.T.); triniac@cyceron.fr (H.T.); bardou@cyceron.fr (I.B.); ali@cyceron.fr (C.A.); agin@cyceron.fr (V.A.); vivien@cyceron.fr (D.V.); 2 University of Nantes, CHU Nantes, Inserm UMR1232, CNRS ERL6001, SFR Santé, FED 4203, Inserm UMS 016, CNRS UMS 3556, CRCINA, Impact Platform, 44200 Nantes, France; Mike.Maillasson@univ-nantes.fr; 3 Candiolo Cancer Institute IRCCS-FPO, Candiolo, 10060 Turin, Italy; tiziana.crepaldi@unito.it (T.C.); pcomoglio@gmail.com (P.C.); 4 Faculty of Biology, Medicine and Health, University of Manchester, Oxford Rd, Manchester M13 9PL, UK; lemarchand@cyceron.fr; 5 Department of Clinical Research, Caen-Normandie University Hospital, CHU, Avenue de la Côte de Nacre, 14000 Caen, France

**Keywords:** neuronal death, tissue-type plasminogen activator, NMDA receptor, MET receptor

## Abstract

Tissue-type plasminogen activator (tPA) plays roles in the development and the plasticity of the nervous system. Here, we demonstrate in neurons, that by opposition to the single chain form (sc-tPA), the two-chains form of tPA (tc-tPA) activates the MET receptor, leading to the recruitment of *N*-Methyl-d-Aspartate receptors (NMDARs) and to the endocytosis and proteasome-dependent degradation of NMDARs containing the GluN2B subunit. Accordingly, tc-tPA down-regulated GluN2B-NMDAR-driven signalling, a process prevented by blockers of HGFR/MET and mimicked by its agonists, leading to a modulation of neuronal death. Thus, our present study unmasks a new mechanism of action of tPA, with its two-chains form mediating a crosstalk between MET and the GluN2B subunit of NMDARs to control neuronal survival.

## 1. Introduction

Tissue-type plasminogen activator (tPA) is a widely expressed protease of the central nervous system [1,2]. Its initially described activity was its ability to activate plasminogen into plasmin [3]. tPA is synthesized and released as a single-chain form (sc-tPA) that is processed into a two-chains form (tc-tPA) within the circulation, or in the extracellular space [4], both forms displaying proteolytic activity [5]. Extending its functions above the conversion of plasminogen into plasmin, tPA interferes with a variety of neuronal receptors, including *N*-Methyl-d-Aspartate receptors (NMDARs) [6]. Interestingly, sc-tPA was reported to promote NMDARs calcium and ERK signalling, and comparatively, tc-tPA was reported to decrease NMDARs’ signalling and death [7].

Hepatocyte growth factor (HGF), and its tyrosine kinase receptor MET, are also present in the central nervous system [8,9]. Pro-HGF is converted into its active form by an extracellular serine protease-dependent proteolytic processing [10]. It has also been reported that in vitro both urokinase PA (uPA) and tPA, as well as factor XII, are able to activate pro-HGF [11,12,13]. In cultured neuronal cells, HGF enhances neurite extension and branching, maturation of the dendritic spine and membrane translocation of NMDARs [14,15]. HGF induces phosphorylation of the GluN2B subunit of NMDARs [15] and protects neurons from aging-related cell death in culture [16] and ischemia [17,18]. Interestingly, HGF enhances NMDA currents and synaptic plasticity in the hippocampus by activating long-term potentiation (LTP) of the CA1 region in vitro [19]. Accordingly, the HGF–MET axis contributes to the activity-dependent regulation of physiological learning and memory performances in the adult brain [20], at least partially by interacting with the NMDARs [15].

Since the pioneering work of Tsirka and colleagues in 1995 [21], a large number of studies have reported a pro-neurotoxic effect of tPA on NMDAR-mediated excitotoxicity [6,22]. However, it is now well established that tPA can promote both neuronal survival and death [23]. For example, although tPA increases NMDARs signalling and its subsequent excitotoxicity [6], it protects the postsynaptic density from the deleterious effects of ischemic injury [24]. These results are consistent with more recent findings showing that tPA is also able to decrease both deleterious endoplasmic reticulum stress [25] and autophagy-mediated [26] neuronal death. However, several questions related to the influence of tPA on neuronal survival and death still remain to be elucidated.

Moreover, tPA exists under two forms: a secreted single-chain form (sc-tPA), that can be processed into a two-chain form (tc-tPA) by plasmin or kallikreins [27]. In the circulation, it is believed that sc-tPA is responsible for the initiation of fibrinolysis by interacting with fibrin through its finger domain to activate locally plasminogen into active plasmin. Plasmin would then generate tc-tPA. PAI-1, an inhibitor of tPA, then competes with the tc-tPA by binding to its kringle-2 domain, and would thus regulate excess fibrinolysis [28]. In the brain parenchyma, it is harder to decipher the role and the proportion of the two isoforms of tPA. We previously show that the sc-tPA was responsible for the activation of NMDAR [29], however no specific action of the tc-tPA on NMDAR has been identified so far.

In the present study, we found that among the two forms of tPA, only the tc-tPA activates neuronal MET independently from the conversion of pro-HGF into active HGF, leading to a proteasome-dependent down-regulation of the GluN2B subunit-containing NMDARs, reduced subsequent NMDARs’ signalling, and no promotion of excitotoxicity. Comparatively, sc-tPA promotes NMDAR signalling, independently from MET, leading to an increased excitotoxic neuronal death. Altogether, these data propose that both forms of tPA contribute differentially to neuronal survival and death, and the tc-tPA promotes a crosstalk between NMDARs and MET.

## 2. Results

### 2.1. tPA Isoforms (sc- and tc-tPA) Differentially Regulate Neuronal Activation of MET

**Figure 1:**
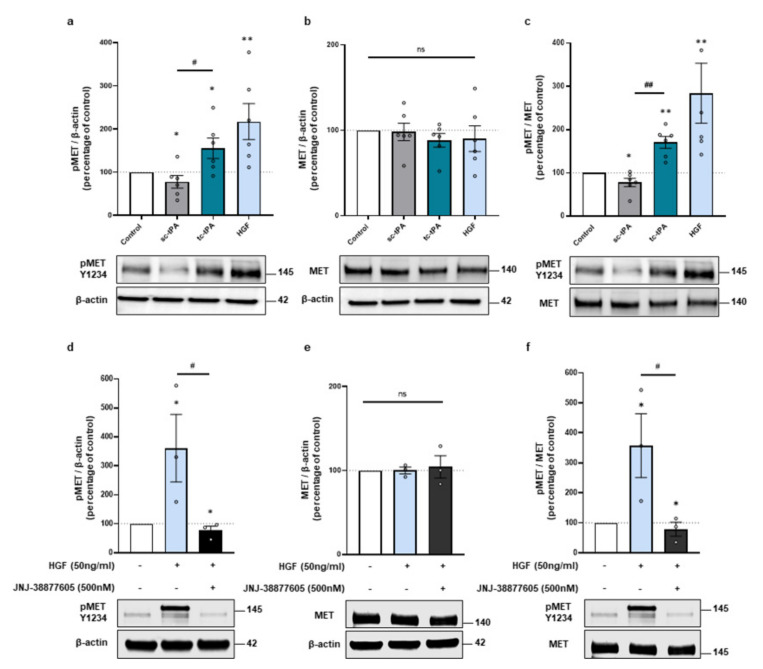
The tc-tPA activates MET receptors. (**a**–**c**), Representative western blots against phospho-MET Y1234 and total MET and quantification from primary cultures of cortical neurons (12–13 DIV) subjected for 1 h to either sc-tPA, tc-tPA (300 nM) or HGF (50 ng/mL). (**a**) Quantification of corresponding ratio of phospho-MET on actin. (**b**) Quantification of corresponding ratio of total MET on actin. (**c**) Quantification of corresponding ratio of phospho-MET on total MET. (**d**–**f**) Representative western blot against phospho-MET Y1234 and total MET and quantification from primary cultures of cortical neurons (12-13 DIV) subjected for 1 h to either HGF (50 ng/mL) either alone or with JNJ-38877605 (500 nM). (**d**) Quantification of corresponding ratio of phospho-MET on actin. (**e**) Quantification of corresponding ratio of total MET on actin. (**f**) Quantification of corresponding ratio of phospho-MET on total MET. Data are represented as mean ± SEM; *n* = 6 (**a**–**c**) and *n* = 3 (**d**–**f**); * *p* < 0.05, ** *p* < 0.01 indicate significantly different from the corresponding control by Mann–Whitney test; ^#^ *p* < 0.05, ^##^ *p* < 0.01 indicate significantly different between treatments with Mann–Whitney test; ns: not significant.

Because the two forms of tPA, sc- and tc-tPA, display differential neuronal functions [7,29], we compared their effects on MET signalling. tc-tPA was prepared from sc-tPA incubated with plasmin. The mixture was then purified with aprotinin-coupled Sepharose 4B at room temperature to eliminate traces of free plasmin. When tc-tPA is added in the media of cortical neurons (1 h, 300 nM at 12-13 DIV) it induced the phosphorylation of MET (+156% of phosphoMET/β-actin, *p* = 0.0476; +171% of phospho/total, *p* = 0.0022; *n* = 6 from six independent treated cultures). In opposition, sc-tPA (1 h, 300 nM) slightly decreased it (−22% of phosphoMET/actin, *p* = 0.0476; −22% of phospho/total, *p* = 0.0476; *n* = 6 from six independent treated cultures) (Figure 1a–c). Control experiments confirmed MET activation upon HGF exposure (+217% of phosphoMET/β-actin, *p* = 0.0022; +284% of phospho/total, *p* = 0.0022; *n* = 6 from six independent treated cultures) (Figure 1a–c). As expected, the specific inhibitor of MET, JNJ-38877605, reversed HGF-induced MET phosphorylation (−68.15% compared to HGF of phosphoMET/β-actin, *p* = 0.0495, −69.02% compared to HGF of phospho/total, *p* = 0.0495; *n* = 3 from three independent experiments (Figure 1d–f). Of note, the expression of total MET did not vary with sc-, tc-tPA, or HGF treatments (98%, 88%, 90%, 104% of control for sc-tPA, tc-tPA, HGF, and HGF + JNJ, respectively, ns = non-significant; *n* = 6 and *n* = 3 for HGF + JNJ) (Figure 1b,e). It has been reported that tPA can cleave pro-HGF into active HGF [11]. Here we tested in vitro, with recombinant protein, if the two forms of tPA were capable to induce the conversion of pro-HGF into HGF. We found that both forms of tPA are inefficient for activating the Pro-HGF; comparatively, FXII as a positive control does (Supplementary Figure S1a). We then tested if tPA could directly bind to MET by using Surface Resonance Plasmonic (SPR) as described in the methods section (Supplementary Figure S1b). Increasing doses of sc-tPA, tc-tPA, and HGF were used on immobilized MET receptors. HGF bound to MET in a dose dependent manner with a Kd = 2.5 × 10^−8^ M as expected. However, our data are inconsistent with the fact that either sc-tPA or tc-tPA could trigger a biological activity through a direct interaction with this receptor. Thus, up until now, we have failed to identify exactly how tc-tPA can activate MET.

### 2.2. MET and NMDA Receptors form Complexes at the Neuronal Surface

**Figure 2:**
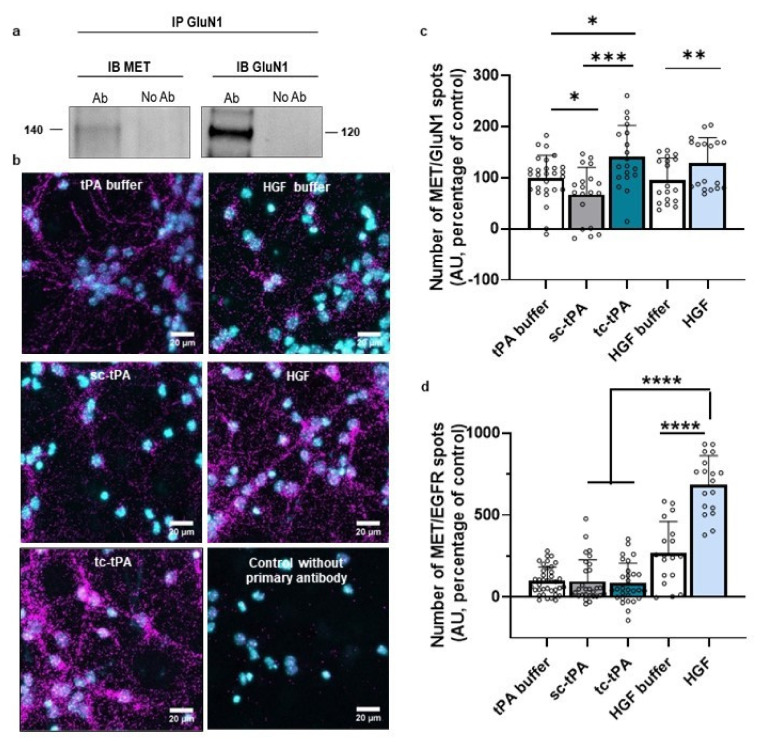
NMDAR and MET form complexes at the neuronal surface modulating by sc-tPA, tc-tPA and HGF. (**a**) Immunoprecipitations with GluN1 antibody revealed by western blot against either MET or GluN1 on primary cultures of cortical neurons (representative blot of three independent experiments). (**b**) Representative confocal microscopy images of PLA staining on primary cultures of cortical neurons treated with tPA buffer, HGF buffer, sc-tPA (300 nM), tc-tPA (300 nM) or recombinant HGF (50 ng/mL) for 45 min. Magenta fluorescent profiles represent regions of PLA signal amplifications denoting MET and NMDAR proximity. (**c**) Quantitative analysis of MET–NMDAR PLA signals normalized to tPA buffer condition. *N* = 18 concerning HGF buffer and HGF conditions, *N* = 19 for sc-tPA (300 nM) and tc-tPA (300 nM) conditions and, *N* = 28 for tPA buffer from four independent cultures. (**d**) Quantitative analysis of MET–EGFR PLA signals normalized to tPA buffer condition. *N* = 18 concerning HGF buffer and HGF conditions, *N* = 29 for sc-tPA (300 nM), tc-tPA (300 nM) conditions, and *N* = 29 for tPA buffer condition, from four independent cultures. Data are represented as mean ± SEM; * *p* < 0.05, ** *p* < 0.01, *** *p* < 0.001 and **** *p* < 0.0001 indicate significantly different from the corresponding control by Mann–Whitney tests.

tPA has already been intensively described as interfering with NMDARs’ signalling [6]. Interestingly, the two forms of tPA have opposite effects on NMDARs’ signalling: although sc-tPA promotes it by increasing calcium influx, tc-tPA decreases it [29]. We investigated whether MET and NMDARs could form complexes by using co-immunoprecipitation assays. The GluN1 subunit co-immunoprecipitated with MET receptor (Figure 2a, representative blots of three independent experiments), confirming the presence of MET–NMDARs complexes under basal conditions. We also performed proximity ligation assay (PLA) to determine whether MET–NMDARs complexes were present at the neuronal surface. PLA performed in cultured neurons showed MET–NMDARs complexes (GluN1/MET) (Figure 2b, representative pictures of 4 independent neuronal cultures). There was virtually no PLA signal in the absence of either primary antibody or the PLA probe. Next, we investigated whether treatment with sc- or tc-tPA influenced the formation of MET–NMDARs complexes. PLA showed that tc-tPA increased the number of MET–NMDARs complexes at the cell surface (+41%, *p* = 0.0211 when compared to tPA buffer condition) whereas sc-tPA decreased the number of MET–NMDARs complexes (−33.2%, *p* < 0.05 when compared to tPA buffer condition; *n* = 28 for tPA buffer, *n* = 19 for sc- and tc-tPA (300 nM) from four independent neuronal cultures; Figure 2b,c). Control experiments with HGF also increased the formation of MET–NMDARs complexes (+128%, *p* = 0.0096 when compared to HGF buffer, *n* = 18 for both HGF buffer and HGF from four independent neuronal cultures; Figure 4b,c). As a positive control, we also performed PLA experiments to determine MET–EGFR complexes. The number of MET–EGFR complexes was not modified when sc- or tc-tPA were added in the culture medium (*p* < 0.05; compared to tPA buffer condition; *n* = 38 for tPA buffer, *n* = 29 for sc-tPA and tc-tPA (300 nM) from four independent neuronal cultures; Figure 2d). Moreover, MET–EGFR complexes were significantly increased in the presence of HGF (+155.62%; *p* < 0.0001 when compared to HGF buffer; *n* = 18 for both HGF buffer and HGF from four independent neuronal cultures; Figure 2d).

### 2.3. tc-tPA Dependent MET Signalling Promotes the Endocytosis and Degradation of Neuronal GluN2B-Containing NMDA Receptors

**Figure 3:**
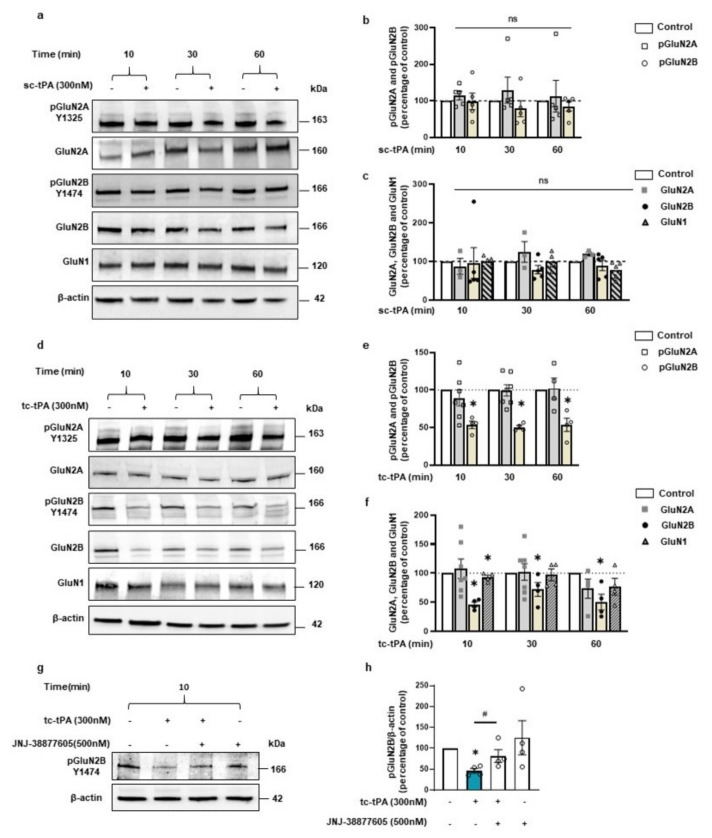
The tc-tPA induces a decrease in GluN2B phosphorylation (Y1474) and total GluN2B through MET receptor. (**a**) Representative western blot and (**b**) corresponding density of the band for pGluN2A (Y1325) and pGluN2B (Y1474) of cultured cortical neurons incubated with sc-tPA (300 nM for 10–30–60 min; *n* = 5 independent experiments). (**c**) Corresponding density of the band for total GluN2A, total GluN2B and total GluN1 (*n* = 5 independent experiments for GluN2B and GluN1, *n* = 3 for GluN2A). (**d**) Representative western blot (**e**) and corresponding density of the band for pGluN2A (Y1325) and pGluN2B (Y1474) of cultured cortical neurons incubated with tc-tPA (300 nM for 10–30–60 min; *n* = 4 independent experiments for GluN2B (Y1474) and *n* = 7 for pGluN2A (Y1325) at 10–30 min and *n* = 4 at 60 min). (**f**) Corresponding density of the band for total GluN2A, total GluN2B and total GluN1 (*n* = 4 independent experiments for GluN2B and GluN1, *n* = 7 for GluN2A at 10–30 min and *n* = 4 at 60 min). (**g**) Representative western blot and (**h**) corresponding density of the band for pGluN2B (Y1474) after treatment on cortical neurons with tc-tPA (300 nM) for 10 min alone or in combination with JNJ-38877605 (500 nM). (*n* = 4 independent experiments). All data are represented as mean ± SEM; * *p* < 0.05, indicate significantly different from the corresponding control by Mann–Whitney test. ^#^ *p* < 0.05 indicate significantly different between treatments by Mann–Whitney test. ns = non-significant.

**Figure 4:**
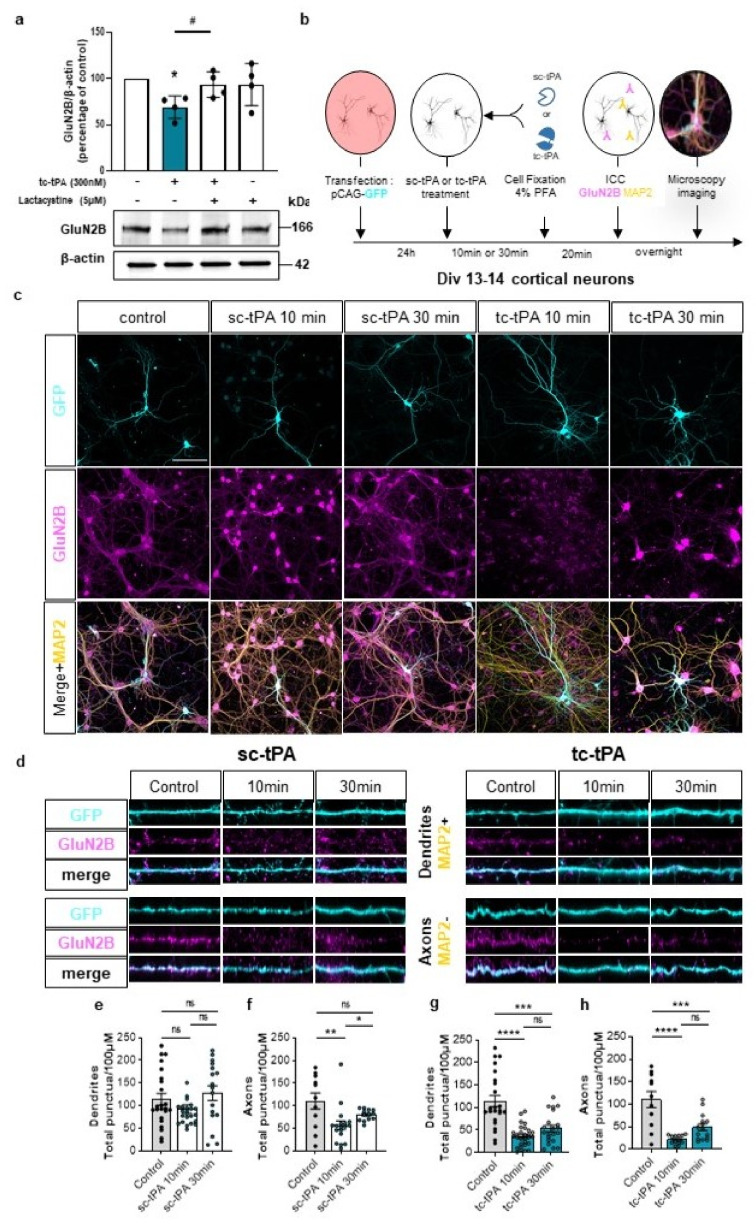
The tc-tPA induces an early endocytosis and degradation of GluN2B. (**a**) Representative western blot and corresponding density for GluN2B of 13 DIV neurons pre-treated or not for 2 h with Lactacystin (5 µM), followed by a 10 min treatment with tc-tPA (300 nM) (*n* = 4 independent experiments). All data are represented as mean ± SEM; * *p* < 0.05 indicate significantly different from the corresponding control by Mann–Whitney test. ^#^ *p* < 0.05 indicate significantly different between treatments by Mann–Whitney test. (**b**) Timeline of the immunocytochemistry experiments. (**c**) Representative images of 13 DIV cortical neurons treated or not with sc- or tc-tPA for 10 or 30 min showing the whole neuronal body in blue (GFP), GluN2B (magenta) and the dendrites in yellow (MAP2). (**d**) Representative images of dendrites and axons used for analyses under different conditions: control, sc-tPA (300 nM during 10 min or 30 min) or tc-tPA (300 nM during 10 min or 30 min). GluN2B puncta/100 µM in dendrites (**e**,**g**) and in axons (**f**,**h**) under sc-tPA (**e**,**f**) or tc-tPA (**g**,**h**) treatments (300 nM for 10 or 30 min) ((**e**), Control: *n* = 23 dendrites, *N* = 13 neurons; tc-tPA 10 min: *n* = 24 dendrites, *N* = 13 neurons; tc-tPA 30 min: *n* = 17 dendrites, *N* = 12 neurons; (**f**), Control: *n* = 11 axons, *N* = 11 neurons; tc-tPA 10 min: *n* = 18 axons, *N* = 18 neurons; tc-tPA 30 min: *n* = 13 axons, *N* = 13 neurons; (**g**), Control: *n* = 23 dendrites, *N* = 16 neurons; tc-tPA 10 min: *n* = 29 dendrites, *N* = 13 neurons; tc-tPA30. min: *n* = 21 dendrites, *N* = 15 neurons; (**h**), Control: *n* = 11 axons, *N* = 11 neurons; tc-tPA 10 min: *n* = 13 axons, *N* = 13 neurons; tc-tPA 30 min: *n* = 15 axons, *N* = 15 neurons). A Shapiro–Wilk test is used to test the normality of data distribution. (**e**,**f**) Kruskal–Wallis’s test followed by a Dunn’s multiple comparisons test were used. (**g**,**h**) One-way ANOVA and a Tukey’s post hoc test were used. Scale bar: 100 µm (**c**). * *p* < 0.05, ** *p* < 0.01, *** *p* < 0.005, **** *p* < 0.0001, ns: not significant.

We then wondered if the effect of tc-tPA on MET–NMDAR complexes could affect NMDAR signalling. Sc-tPA had no effect on GluN2B phosphorylation (Figure 3a and corresponding densitometry Figure 3b) (−1.2%, −21.4%, −15.2% of phospho-GluN2B compared to control at 10, 30, and 60 min, respectively, *p*: ns; *n* = 5 from five independent neuronal cultures), neither on total GluN2B (Figure 3a and corresponding densitometry Figure 3c) (−4%, −21.37%, −10.35% of total GluN2B compared to control at 10, 30, and 60 min, respectively, *p*: ns; *n* = 5 from five independent neuronal cultures). By contrast, tc-tPA induced an early (from 10 min to 60 min) down-regulation of phospho-GluN2B (Figure 3d and corresponding densitometry Figure 3e) (−44.26%, −53.18%, −46.61% of control at 10, 30, and 60 min, respectively, *p* = 0.0139; *n* = 4 from four independent neuronal cultures) and total GluN2B subunits of NMDARs (Figure 3d and corresponding densitometry Figure 3f) (−53.69%, −27.84%, −49.54% of control of total GluN2B at 10, 30, and 60 min, respectively, *p* = 0.0139; *n* = 4 from four independent neuronal cultures). Levels of both phosphorylated and total GluN2A subunits of NMDARs were not affected by tPA treatments (Figure 3a–f). A moderate but significant reduction of the common GluN1 subunit of NMDARs was also observed 10 min after tc-tPA exposure but was not significant at 30 and 60 min (Figure 3d,f) (−9.46%, −2.78%, −21% of GluN1 at 10, 30, and 60 min, respectively, *p* = 0.0139; *n* = 4 from four independent neuronal cultures). As expected, sc-tPA did not influence GluN1 expression (Figure 3a,c) (−0.9%, −0.8%, −22.2% of GluN1 at 10, 30, and 60 min, respectively, *p*: ns; *n* = 5 from five independent neuronal cultures). Importantly, the tc-tPA-induced down-regulation of GluN2B subunit-containing NMDARs was reversed by the presence of the specific inhibitor of MET phosphorylation, JNJ-38877605 (Figure 3g,h) (+35.2% of phospho-GluN2B compared to tc-tPA, *p* = 0.0433; +4.6% of total GluN2B compared to tc-tPA, ns; *n* = 4 from four independent experiments for phospho-GluN2B and total GluN2B). Altogether, these data suggest a tc-tPA dependent endocytosis and degradation of GluN2B-containing NMDARs, mediated by MET signalling. In order to validate this hypothesis, similar experiments were performed in the presence of Lactacystin (5 µM), an inhibitor of the proteasome (Figure 4a). Our data revealed that the tc-tPA-dependent degradation of GluN2B-containing NMDARs (−30.95% of total GluN2B compared to control, *p* = 0.0286) (Figure 4a) was reversed by a pre-treatment with Lactacystin (2 h before tc-tPA, Lactacystin and tc-tPA: +24.49% of total GluN2B compared to tc-tPA alone, *p* = 0.0286; *n* = 4 from four independent experiments) (Figure 4a). We confirmed this by immunocytochemistry of total GluN2B in permeabilized conditions with either sc- (Figure 4c–f) or tc-tPA (Figure 4c,d,g,h) treatment. In dendrites, tc-tPA decreased total GluN2B at 10 min (−68.3% compared to control, *p* < 0.0001) and 30 min (−52.92 % compared to control, *p* = 0.0001), whereas sc-tPA had no effect (*n* = 23 dendrites for control, *n* = 41 dendrites for sc-tPA and *n* = 50 dendrites for tc-tPA, from around 66 neurons from 13 independent neuronal cultures). In axons, tc-tPA decreased total GluN2B at 10 min (−80.3% compared to control, *p* < 0.0001) and 30 min (−55.72% compared to control, *p* = 0.0003), while sc-tPA had only a slight effect at 10 min only (−48.628% compared to control for 10 min, *p* < 0.0094 and NS at 30 min) (*n* = 11 axons for control, *n* = 31 axons for sc-tPA and *n* = 28 axons for tc-tPA from around 70 neurons from seven independent neuronal cultures). This is possibly due to conversion of sc-tPA into tc-tPA in the corresponding bathing media.

### 2.4. tc-tPA-Dependent MET Signalling Controls NMDA Receptors-Mediated Calcium Influx

**Figure 5:**
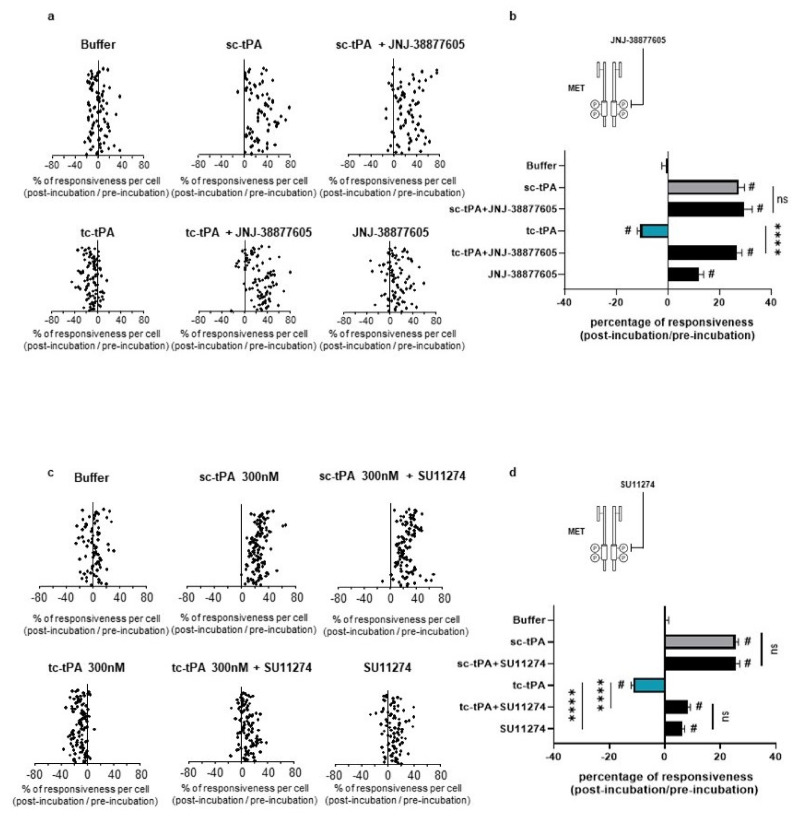
Inhibition of the HGF–MET axis counteracts the tc-tPA-dependent decrease in NMDAR-mediated neuronal calcium influx. Calcium video imaging performed on primary cultures of cortical neurons (13 DIV). After control NMDA stimulations (25 mM) used as baseline, neurons were incubated for 45 min with (**a**,**b**) JNJ-38877605 buffer + tPA buffer (control, *n* = 88 cells), JNJ-38877605 (500 nM; *n* = 91 cells), sc-tPA (300 nM; *n* = 77 cells) and tc-tPA (300 nM; *n* = 98 cells) alone or combined with JNJ-38877605 (*n* = 82 cells and *n* = 98 cells, respectively); or (**c**,**d**) SU11274 buffer + tPA buffer (control, *n* = 78 cells), SU11274 (2 µM; *n* = 93 cells), sc-tPA (300 nM; *n* = 118 cells) and tc-tPA (300 nM; *n* = 108 cells) alone or in combination (*n* = 111 cells and *n* = 121 cells, respectively). Each dot represents one cell. (**b**,**d**) Percentage of stimulation or inhibition after incubation were calculated for each individual cell and reported as the percentages of responsiveness for each group (*n* = 3 independent experiments, mean ± SEM). ^#^ indicates significant difference for the comparison of pre- and post-incubation responses by Wilcoxon signed-rank test (*p* < 0.0001). **** indicates significantly different from the corresponding control by Kruskal–Wallis and Mann–Whitney tests (*p* < 0.0001). ns: not significant.

**Figure 6:**
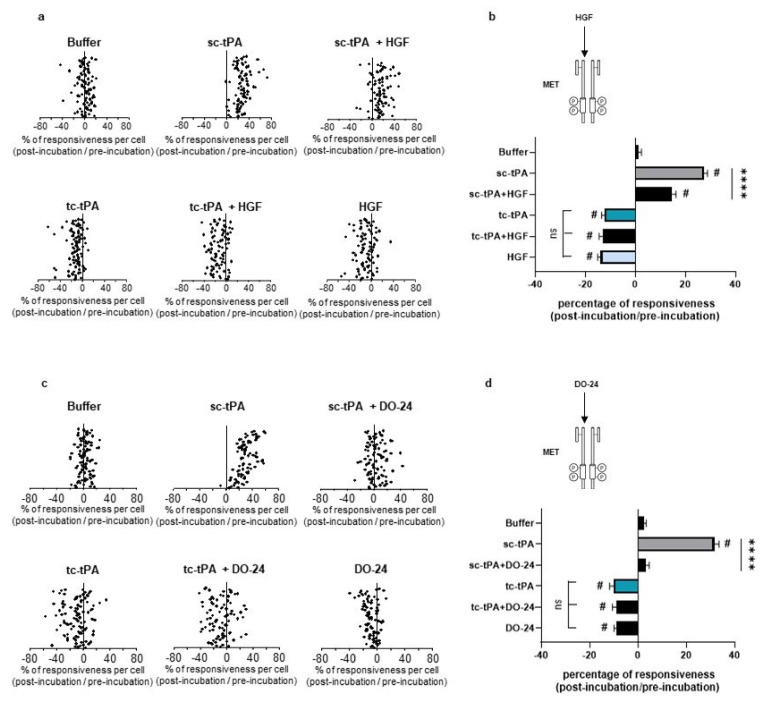
Activation of the HGF–MET axis counteracts the sc-tPA-dependent increase in NMDAR-mediated neuronal calcium influx. Calcium video imaging performed on primary cultures of cortical neurons (13 DIV). After control NMDA stimulations (25 mM) used as baseline, neurons were incubated for 45 min with (**a**,**b**) HGF buffer + tPA buffer (control, *n* = 96 cells), rHGF (50 ng/mL; *n* = 91 cells), sc-tPA (300 nM; *n* = 93 cells) and tc-tPA (300 nM; *n* = 100 cells) alone or combined with rHGF (*n* = 89 cells and *n* = 88, respectively); or (**c**,**d**) DO-24 buffer + tPA buffer (control, *n* = 95 cells), DO-24 (50 ng/mL; *n* = 88 cells), sc-tPA (300 nM; *n* = 86 cells) and tc-tPA (300 nM; *n* = 100 cells) alone or combined with DO-24 (*n* = 94 cells and *n* = 86 cells, respectively). Each dot represents one cell. (**b**,**d**) Percentage of stimulation or inhibition after incubation were calculated for each individual cell and reported as the percentages of responsiveness for each group (*n* = 3 independent experiments, mean ± SEM). ^#^ indicates significant difference for the comparison of pre- and post-incubation responses by Wilcoxon signed-rank test (*p* < 0.0001). **** indicates significantly different from the corresponding control by Kruskal–Wallis and Mann–Whitney tests (*p* < 0.0001).

As previously reported [7], addition of tc-tPA to the neuronal culture media decreased the NMDA-induced calcium influx (−10.66%, *p* < 0.001; *n* = 98 from three independent neuronal cultures), while sc-tPA treatment was stimulatory (+27.29%, *p* < 0.001; *n* = 77 from three independent neuronal cultures) (Figure 5a; corresponding mean Figure 5b). Interestingly, the specific MET inhibitor, JNJ-38877605, reversed the inhibitory effect of tc-tPA (−10.66%, for tc-tPA alone versus +26.6% for tc-tPA + JNJ-38877605, *p* < 0.001; *n* = 98 from three independent neuronal cultures) (Figure 5a and corresponding mean Figure 5b). Similar results were obtained with another MET inhibitor, SU11274, which competes with the ATP binding site within the MET activation loop [30] (−11.24% for tc-tPA alone versus +8.30% for tc-tPA + SU11274, *p* < 0.0001) (Figure 5c and corresponding mean Figure 5d). Accordingly, addition of HGF (Figure 6a and corresponding mean Figure 6b) or DO-24 (a monoclonal antibody agonist of MET) (Figure 6c and corresponding mean Figure 6d) did not affect the action of tc-tPA, but reduced the stimulatory effect of sc-tPA on NMDARs’ signalling (+27.44% for sc-tPA alone versus +14.69% for sc-tPA + HGF, *p* < 0.001; *n* = 93 for sc-tPA and *n* = 89 for sc-tPA and HGF from three independent neuronal cultures; Figure 6a,b; and +31.57% for sc-tPA alone versus +3.30% for sc-tPA + DO24, *p* < 0.001; *n* = 86 for sc-tPA and *n* = 94 for sc-tPA and DO-24 from three independent neuronal cultures; Figure 6c,d). Altogether, these data demonstrate dual effects of tPA isoforms, with sc-tPA promoting NMDARs’ signalling while tc-tPA reduced it. Similarly, HGF prevented the potentiation of NMDA-mediated calcium influx by sc-tPA and the MET agonist DO-24 antibody completely blocked the stimulatory effect of sc-tPA (Figure 6c,d). The blockage of MET activation by JNJ-38877605 (Figure 5a,b) or SU11274 (Figure 5c,d) did not affect the potentiating effect of sc-tPA. We also combined sc-tPA with increasing concentrations of tc-tPA and evaluated the corresponding NMDA-induced neuronal calcium influx (Supplementary Figure S2a,b). In agreement with our above data, tc-tPA counteracted the effect of sc-tPA on NMDAR signalling (−22.26% at 100 nM of tc-tPA, −32.74% at 200 nM of tc-tPA and −33.20% at 300 nM of tc-tPA, *p* < 0.0001).

### 2.5. tc-tPA Protects from NMDA-Mediated Excitotoxicity through MET Activation

**Figure 7:**
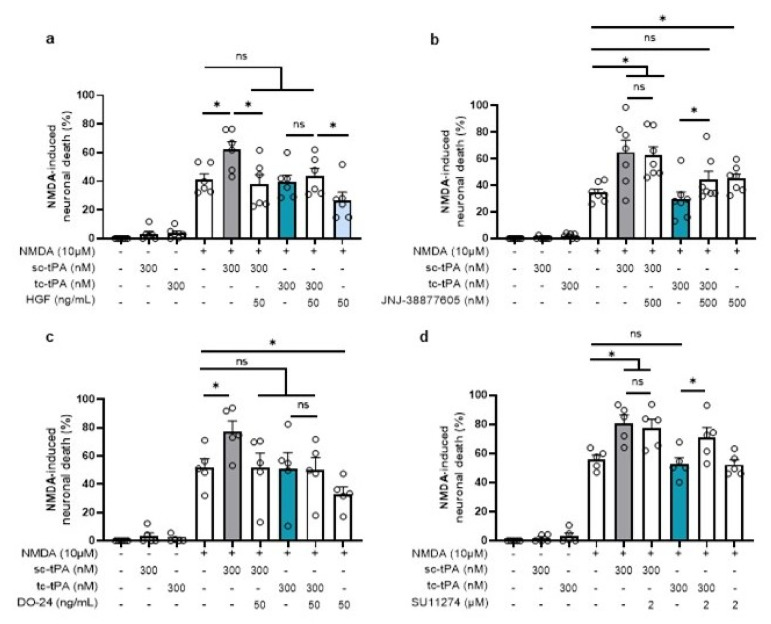
Inhibition or activation of MET affects the potentiation of NMDA-mediated neuronal death. Neuronal death was assessed on primary cultured cortical neurons (12–14 DIV) by measuring LDH release in the bathing media after 24 h exposure to NMDA (10 µM), sc-tPA (300 nM) and tc-tPA (300 nM) alone or combined with (**a**) rHGF (50 ng/mL; *n* = 6, mean ± SEM), (**b**) JNJ-38877605 (500 nM; *n* = 7, mean ± SEM), (**c**) DO-24 (50 ng/mL; *n* = 5, mean ± SEM), (**d**) SU11274 (2 µM; *n* = 5, mean ± SEM). N = 5 independent experiments; * indicates significantly different from the corresponding control by Kruskal–Wallis and Mann–Whitney tests (*p* < 0.05). ns: not significant.

We then investigated whether the tc-tPA dependent MET signalling also influenced the NMDAR-mediated neuronal death. As previously reported [29], sc-tPA promoted NMDA-induced excitotoxicity (+20.86%, *p* = 0.025) and tc-tPA did not (−1.69%, ns) (Figure 7). In agreement with the above data, inhibition of MET by the JNJ-38877605 (Figure 7b) and the SU11274 (Figure 7d), converted the neuroprotective effect of tc-tPA to a pro-excitotoxic effect (+14.46% for tc-tPA + JNJ-38877605, *p* =0.0253 (*n* = 7 from seven independent experiments); +18.81% for tc-tPA + SU11274, *p* = 0.0472; *n* = 5 from five independent experiments). Thus, sc-tPA potentiated NMDA-induced excitotoxicity was prevented by co-application of either rHGF (Figure 7a) (+20.86% for sc-tPA alone versus −3.73% for sc-tPA + rHGF, *p* = 0.0374; *n* = 6 from six independent experiments) or by the agonist MET antibody DO-24 (Figure 7c) (+25.31% for sc-tPA alone versus −0.73% for sc-tPA + DO-24, *p* = 0.0472; *n* = 5 from five independent experiments).

## 3. Discussion

We provide here a novel mechanism of the actions of tPA in neurons, with the first demonstration that the serine protease tPA, especially its two chain form (tc-tPA), contributes to a neuronal crosstalk between MET and GluN2B subunit-containing NMDARs. We then demonstrate that this mechanism relies on the formation of proximal complexes between MET and NMDARs at the neuronal surface that leads to a proteasomal degradation, subsequent down-regulation of the total number of surface GluN2B-containing NMDARs and finally to a reduced NMDARs’ signalling and neuroprotection in vitro.

Several studies demonstrated the physical interaction between NMDAR subunits and other receptors, including Dopamine D1 receptor (D1R) [31], VEGF receptor (VEGFR2) [32], and Ephrin-B2 [33]. Ligand-dependent activation of VEGFR2 induces a rapid redistribution of GluN2B at synaptic sites, influencing the consolidation of emotional memory [32]. Here, we provide evidence that tc-tPA leads to a connection between NMDARs and MET at the neuronal surface, and that the dynamic of these interactions plays a critical role in the control of NMDARs’ signalling and neuronal fate. These data are in agreement with recent reports supporting that genes encoding for NMDARs subunits are part of the interactome of MET, with roles in the modulation of synapses formation [34] and of neurodevelopmental disorders such as ASD [35].

Such as all members of the plasminogen activator family, the secreted sc-tPA is processed into tc-tPA by plasmin or kallikrein [36]. However, sc-tPA is an unusual zymogen that does not require proteolytic processing to be activated, but relies on the presence of an allosteric regulator, such as fibrin [5]. It has been reported that sc-tPA, but not tc-tPA, promotes NMDARs’ signalling and neurotoxicity in cortical neurons [7,29]. Here, we provide a mechanistic explanation of this differential function of the two forms of tPA. We demonstrate that, although tc-tPA prompts the formation of proximal complexes between MET and NMDARs, sc-tPA promotes their disruption. Interestingly, we also demonstrated that tc-tPA enhances MET signalling, leading to an increased endocytosis and degradation of GluN2B-containing NMDARs and subsequent down-regulation of NMDARs-mediated signalling and excitotoxicity. Notably, recombinant HGF (or the MET agonist DO-24 antibody, mimicking the ligand), completely prevents sc-tPA-induced NMDAR signalling and neurotoxicity in vitro.

tPA is known to mediate signalling through a set of receptors and binding proteins including the low density lipoprotein related receptors (LRPs), annexin-II, epidermal growth factor receptor (EGFR) and NMDARs, among others [37]. Although there is a consensus about the capacity of tPA to modulate NMDARs’ signalling and functions, some opposite results are reported in the literature [6,38]. When interacting with the GluN2A subunit, tPA activates either the Akt/mTOR/p70S6K/HIF1α signalling pathway, leading to neuroprotection and to an increase in glucose uptake [39] or the Erk1/2/CREB/ATF3 signalling pathway to decrease excitotoxicity [40]. tPA was also reported to interact [41] and possibly cleave the GluN2B subunit of NMDAR to change its pharmacological properties. GluN2B-dependent effects were associated with roles of tPA in acute stress [42] and on seizures induced by alcohol withdrawal [41]. The sc-tPA was reported to interact with the GluN1 subunit of NMDARs either directly [6] or through LRP1 [43] to promote NMDARs’ signalling and excitotoxicity. Our study may reconcile all these data. Indeed, we evidence here that sc-tPA and tc-tPA differentially influence NMDARs’ signalling, with sc-tPA leading, as previously reported [29], to an activation of NMDARs, due to its ability to bind the common GluN1 subunit [6], and tc-tPA, leading to a specific down-regulation of GluN2B-containing NMDARs, a mechanism dependent on MET activation.

NMDARs are mainly found on the postsynaptic membrane at excitatory synapses but are also present at extra-synaptic positions [44]. It is believed that their localization at the synapse might explain their roles in the neuronal fate. Indeed, extrasynaptic NMDARs have been reported to be neurotoxic [45], and more importantly, the subunit composition of the receptors, and essentially the GluN2 subunit, is a key determinant of their effect on the neuronal fate, with GluN2B being associated to cell death [46]. This is in line with previous data showing that tPA increases the diffusion of extrasynaptic NMDARs [24] and the current work showing that tc-tPA increases the degradation of GluN2B-containing NMDAR through MET to decrease NMDA-mediated excitotoxicity.

Altogether, our data reveal a new mechanism of action of tPA in the central nervous system, with a differential role of its sc and tc forms, with the tc-tPA capable of activating MET, thus promoting the formation of complexes with GluN2B-containing NMDARs, their endocytosis and proteasomal down-regulation, subsequent reduced signalling, and finally their neurotoxicity.

## 4. Material and Methods

### 4.1. Chemicals

*N*-methyl-d-aspartate (NMDA) was purchased from Tocris (Pittsburgh, PA, USA). Human tPA (Actilyse^®^) was from Boehringer Ingelheim (Paris, France). Dulbecco’s modified Eagle’s medium (DMEM), poly-d-lysine, glutamine, cytosine β-d-arabinoside (Ara-C), glycine, foetal bovine serum, horse serum, Triton X-100, anti-rabbit (A0545) peroxidase antibody, protease inhibitor cocktail (P8340), phosphatase inhibitor cocktail (P5726) and Lactate dehydrogenase (LDH) detection kit and Lactacystin (CAS1258004) were from Sigma-Aldrich (Saint-Quentin Fallavier, France). Pierce BCA Protein Assay Kit (23225) ECL-Plus detection system (32132) and lipofectamine^®^ 2000 reagent was purchased from Thermo Fisher Scientific (Illkrich-Graffenstaden, France).

### 4.2. Preparation of sc-tPA and tc-tPA

Sc-tPA and tc-tPA were prepared from Actilyse^®^ (Boehringer Ingelheim). Sc-tPA was obtained by dialysing the commercial solution in HEPES buffer (4-(2-hydroxyethyl)-1-piperazineethanesulfonic acid, 0.3 M, pH 7.4, Sigma-Aldrich) using dialysis cassettes (Slide-A-Lyzer^®^ 10K; ThermoScientific). Tc-tPA was prepared by overnight incubation of Actilyse^®^ with plasmin-coupled Sepharose 4B at 37 °C, followed by two hours of incubation with aprotinin-coupled Sepharose 4B at room temperature to eliminate traces of free plasmin. Then, tc-tPA was dialyzed in HEPES buffer (0.3 M, pH 7.4, Sigma-Aldrich) using the same dialysis cassettes as for sc-tPA preparation. Finally, sc-tPA (i.e., dialyzed Actilyse^®^) and tc-tPA preparations ratio were controlled by SDS-PAGE (10% polyacrylamide gels) with Coomassie blue staining, and the amydolytic activity has been checked by spectrozyme assay. Only sc-tPA presenting less than 10% of tc-tPA was used.

### 4.3. Neuronal Cell Cultures

Culture of cortical neurons were prepared from foetal mice (embryonic day 14) as previously described [47]. Cortices were dissected and mechanically dissociated in DMEM, seeded on 24 well plates or on dishes previously coated with poly-d-lysine (0.1 mg/mL; Sigma-Aldrich P6407) and laminin (0.02 mg/mL; Life Technologies 23017-015). Cells were cultured in DMEM (Sigma-Aldrich D5671) supplemented with 5% foetal bovine serum (Sigma-Aldrich F9665), 5% horse serum (Sigma-Aldrich H1138) and 2 mM glutamine (Sigma-Aldrich G3156). Cultures were maintained at 37 °C in a humidified 5% CO_2_ atmosphere. Cytosine β-d-arabinoside (10 μmol/L; Sigma-Aldrich C1768) was added after 3 days in vitro (DIV) to inhibit glial proliferation.

### 4.4. Hypodense Neuronal Cultures

Primary cultures of cortical neurons were prepared from foetal mice (embryonic day 14) as described above. Cortices were dissected and dissociated in DMEM and plated (250,000 cell/mL) on glass bottom microwell dishes (MatTek Corporation, P35G-1.5-14-C, Ashland, MA, USA) earlier coated with poly-d-lysine (0.1 mg/mL) and laminin (0.02 mg/mL). Cells were cultured in Neurobasal Medium supplemented with 0.4 mM of glutamine, 2% B27 supplement 50X, 10% horse serum and penicillin/streptomycin (10,000 IU/mL; 10,000 UG/mL). After 1 h, media were replaced by conditioned medium (Neurobasal Medium supplemented with 0.4 glutamine, 2% B27 supplement 50X and penicillin/streptomycin (10,000 IU/mL; 10,000 UG/mL) obtained from primary cultures of astrocytes. Cultures were maintained at 37 °C in a humidified 5% CO_2_ atmosphere. Cytosine β-d-arabinoside (10 μmol/L; Sigma-Aldrich C1768) was added after 3 DIV. Cultures have been used for neuronal transfection and immunocytochemistry.

### 4.5. Proximity Ligation Assay

The proximity ligation assay was performed using the Duolink In Situ Kit (Olink Bioscience, Uppsala, Sweden) according to the manufacturer’s instructions with the following modifications: cell dishes were treated with buffer, rHGF, sc-tPA and tc-tPA for 1 h, then fixed with paraformaldehyde 4% supplemented with 4% sucrose. PLA probe incubation was 2 h and amplification step was extended to 2 h. Blocking (1 h at room temperature) and primary antibody (overnight at 4 °C) incubations were performed in a 4% bovine serum albumin and 0.2% Triton X-100 solution. Goat anti-GluN1 (1:250; Santa-Cruz Biotechnology sc-1467, Dallas, TX, USA), rabbit anti-MET (1:250; Santa-Cruz Biotechnology sc-161), and rat anti-EGFR (1:250, abcam ab231) were diluted in the blocking solution. The anti-rabbit (+) PLA probe (1:5) along with an anti-rat (−) probe (1:100) were diluted in the blocking solution. A goat anti-rat (Jackson Immuno Research Inc., Suffok, UK) was used to make a probe anti-rat according to the manufacturer’s instructions using the Duolink Probemaker (Olink Bioscience, Uppsala, Sweden). The negative control represents the PLA without primary antibodies. A total of ten stacks of picture (0.40 μm per section) were taken from 10 different areas of every well with a confocal inverted microscope (Leica SP5, Leica, Nanterre, France). Imaris modelling: a “cell function” analysis was performed in order to label neuronal cell bodies, then the “spot” function was used to detect positive PLA puncta prior counting. This procedure was performed blind by using a Z stack-projection from 10 independent areas per condition and per experiment of a total of 4 independent experiments. Concerning the quantification, for each area, the quantity of puncta has been subtracted to the mean quantity of buffer and then data were normalised to the tPA buffer condition.

### 4.6. Immunoblotting

Immunoblottings were performed from treated-cortical neurons (DIV12/13). Cells were dissociated in ice-cold TNT buffer (50 mM Tris-HCl, pH 7.4, 150 mM NaCl, and 0.5% Triton X-100) supplemented with a cocktail of protease and phosphatase inhibitors. Protein quantification was performed according to the BCA method. Proteins (20 μg) were resolved on polyacrylamide gel under denaturing conditions and transferred onto a polyvinylidene difluoride membrane. Membranes were blocked with Tris-buffered saline (10 mM Tris and 200 mM NaCl, pH 7.4) containing 0.1% Tween 20 and 1% BSA for 1 h at room temperature. Blots were incubated overnight with primary antibodies at 4 °C. The primary antibodies used were anti-phospho-MET (Y1234/1235) (Santa Cruz Biotechnology, sc-101736, diluted 1:1000), anti-Met (R&D systems, AF527, diluted 1:1000), anti-HGF (Invitrogen, SBF5, diluted 1:1000), anti-GluN2B (abcam, ab65783, diluted 1:1000), anti-phospho-GluN2B (Y1474) (abcam, ab194923, diluted 1:1000), anti-GluN2A (Y1325) (abcam, ab16646, diluted 1:1000), anti-GluN2A (abcam, ab14596, diluted 1:1000), anti-GluN1 (Santa-Cruz Biotechnology, sc1467, diluted 1:1000), anti-actin (Sigma-Aldrich, A2066, diluted 1:1000). This step was followed by incubation with the appropriate peroxydase-conjugated secondary antibody which were goat anti-rabbit (Sigma, A05445, diluted 1:50,000), rabbit anti-goat (Sigma, A8919, diluted 1:160,000). Immunoblots were revealed with an enhanced chemoluminescence ECL-Plus immunoblotting detection system and imaged in an Image Quant LAS 4000 Device (GE Heathcare, Orsay, France).

### 4.7. Excitotoxic Neuronal Death

Neurons (12-13 DIV) were rinsed three times with serum-free medium (DMEM) supplemented with glycine (20 µM), excitotoxicity was induced by exposure to NMDA (10 μM) in serum-free DMEM supplemented with 20 μM of glycine for twenty-four hours [47]. NMDA was applied alone or in the presence of either sc-tPA (300 nM), tc-tPA (300 nM), recombinant HGF (50 ng/mL; rHGF), DO-24 antibody (50 ng/mL; agonist of MET), JNJ-38877605 (500 nM; antagonists of MET) or SU11274 (2 µM; inhibitor of MET activation) alone or in combination, as mentioned in the legend of the corresponding figures. After twenty-four hours, neuronal death was quantified by measurement of lactate dehydrogenase (LDH) released from damaged cells.

### 4.8. Crossed Immunoprecipitation Assays

Supernatants from TNT buffer (50 mM Tris-HCl, pH 7.4, 150 mM NaCl and 0.5% Triton X-100)-lysed cultured cortical neurons (13 DIV) (100 μg of total proteins) were incubated overnight at 4 °C with an antibody (raised against the GluN1 subunit of NMDAR (1 µg, Santa-cruz Biotechnology, Dallas, USA, sc-1467). This antibody was coupled to protein G-sepharose beads as described by the manufacturer (GE Healthcare, Buc, France) for immunoprecipation procedures, including repeated washes in TNT buffer. Then, immunoprecipitated proteins were separated by 10% SDS-PAGE and immunoblots were revealed with either an antibody raised against total GluN1 (Santa-Cruz Biotechnology, sc-1467, diluted 1:1000) or an antibody raised against total MET (R&D systems, Noyal Châtillon sur Seiche, France, AF527, diluted 1:1000) by following the procedure described above (see «Immunoblotting» section).

### 4.9. Calcium Video Microscopy

Experiments were performed at room temperature on the stage of an inverted calcium microscope (Leica DMI6000B, Nanterre, France) equipped with a 150 W Xenon high stability lamp and a Leica 40×, 1.3 numerical aperture epifluorescence oil immersion objective. Fura-2 ratio images were acquired with a Digital CMOS camera (ORCA-Flash2.8 C11440-10C, Hamamatsu, Japan) and digitized (2048*2048) using Metafluor^®^ 6.1 software (Universal Imaging Corporation). Cultures were transferred into a serum-free HEPES-buffered saline solution (HBBSS: NaCl 116 mM, KCl 5.4 mM, CaCl_2_ 1.8 mM, MgSO_4_ 0.8 mM, HEPES 12 mM, NaH_2_PO_4_ 0.34 mM, D-Glucose 5.5 mM, NaHCO_3_ 25 mM, Glycine 10 µM) at DIV 12 and loaded with Fura-2-AM (10 µM; Invitrogen, Life Technologies F1201, Saint Aubin, France) diluted in pluronic acid (Life Technologies P-3000MP) for 45 min at 37 °C. The Ca^2+^ bound form of Fura-2 gets excited at 340 nm, the Ca^2+^ unbound form at 380 nm and both recorded at 510 nm. Neurons were washed with HBBSS flux and NMDA stimulations (2 × 25 µM for 30 s) were applied using a peristaltic pump. Prior to a second run of NMDA stimulations, neurons were incubated for 45 min with either sc-tPA (300 nM), tc-tPA (300 nM), recombinant HGF (50 ng/mL; rHGF; Gibco, Life Technologies PHG0254, Saint Aubin, France), anti-HGF blocking (50 ng/mL; Santa-Cruz Biotechnology sc-1356), DO-24 antibody (50 ng/mL; agonist of MET), JNJ-38877605 (500 nM; antagonist of MET) or SU11274 (2 µM; inhibitor of MET activation; Selleckchem S1080, Houston, TX, USA) or Glunomab^®^ (antagonist of the action of tPA on the GluN1 subunit of NMDAR) alone or in combination. For each cell, the area under curve (AUC) corresponding to the intracellular calcium influx induced by NMDA (ratio 340/380) was calculated before (AUCb) (mean of the two first NMDA stimulations) and after treatment (AUCa) (mean of the two second post-treatment stimulations). We then compared, for each cell, the amount of NMDA-induced calcium influx after treatment with the NMDA-induced calcium influx recorded before treatment. Thus, each cell is its own control with an expression of the modification of the NMDA-induced calcium influx due to the treatment or their excipient performed between the two rounds of stimulations, expressed as a percentage of response to treatments (% of responsiveness). % of responsiveness = [(AUC)a/(AUC)b]*100 with (AUC)a: sum of area under curve of Fura-2 ratio during both NMDA stimulations after treatment and (AUC)b: sum of area under curve of Fura-2 ratio during NMDA stimulations before treatment.

### 4.10. Neuronal Transfection

Transfections were performed at 11 DIV on hypodense neuronal cultures. Neuronal cultures were washed with HEPES and Bicarbonate Buffered Salt Solution (HBBSS; composition in calcium video microscopy section) prior to an 8 h incubation in the presence of pCDNA5-GFP plasmid and lipofectamine^®^ 2000 containing HBBSS. Then, HBBSS was replaced by regular media as described above (hypodense neuronal cultures section).

### 4.11. Immunocytochemistry

Hypodense neuron cultures (13 DIV) were used to visualize neural extensions. Cultures treated with tc-tPA (300 nM) for 10 or 30 min and washed with HBBSS. Neurons were fixed in paraformaldehyde 4% for 20 min at room temperature, washed in PBS (0.1 M) followed by a 1 h blockage in PBS supplemented either with BSA 4% and 0.3% Triton X100 for the permeability conditions or with BSA 4% for the surface experiments. A rabbit anti-GluN2B N-term antibody (Alomone Labs, AGC-003, diluted 1:500) with a chicken anti-MAP2 antibody (abcam, ab5392, diluted 1:8000) were incubated overnight at 4 °C. Neurons were subsequently rinsed 3 times with PBS (0.1 M) and were incubated 1 h and a half at room temperature with a Cy3-conjugated anti-rabbit and an Alexa-647-conjugated anti-chicken secondary antibodies (Jackson Immunoresearch, West Grove, PA, USA, 1:800). Immunocytochemistry were examined with a Leica TCS SP8 confocal/STED microscope 3x microscope with an oil-immersion 40x, 1.44-N.A objective and further processed using ImageJ software (NIH; Bethesda, MD, USA). The number of punctua per 100 µm on processes was calculated with the “pluginTrackmate”.

### 4.12. Surface Plasmon Resonance

SPR experiments were performed on a Biacore T200 (GE Healthcare) at 25 °C. HBS-EP buffer was filtered through a 0.45 μm membrane filter and degassed prior to use. First, recombinant Human HGF R/MET Fc chimera were immobilized on the chip surface by amine coupling. Briefly, recombinant MET receptor was diluted to 20 µg/mL in 5 mM Maleate solution at pH 6.2. The diluted MET protein was soon covalently immobilized to a flow cell of CM5 sensor chip via primary amine group, using standard Amine Coupling Kit. HGF, sc-tpA, tc-tpA were then analysed in a “Single Cycle kinetics” (SCK) models over the MET immobilized chip.

The affinity (KD) and kinetics parameters (ka and kd) of HGF, sc-tpA and tc-tpA over MET were determined by using a series of proteins dilutions in a “Single Cycle Kinetics” (SCK) model. HGF, sc-tpA and tc-tpA, as the analytes, were diluted in HBS-EP buffer with concentrations ranging from 0.6 nM to 50 nM for HGF and from 25 nm to 2000 nM for sc-tpA and tc-tpA, which flowed over the immobilized MET and the obtained response units (Rus) were recorded. The flow rate was at 30 μL/min with 120 s for binding and 600 s for dissociation. Then, the sensor chip surface was regenerated with Ethanolamine 1 M, pH 8.5 for 30 s. The dissociation equilibrium constant, KD, and kinetics parameters, kd and ka, were determined by direct curve fitting of the sensorgrams to a Langmuir 1:1 model of interaction.

### 4.13. Statistics

Data have been analysed with Prism (Graphpad) software. Shapiro–Wilk tests were used to ensure a normal distribution. Comparisons of two data sets were performed using unpaired two-tailed Student’s *t*-test for normally distributed data sets and Mann–Whitney test for non-normally distributed data sets. Comparisons for multiple data sets were performed using one-way analysis of variance with Tukey’s post-hoc test for normally distributed data sets and Kruskal–Wallis test with Dunn’s multiple comparison test for non-normally distributed data sets.

## Supplementary Materials

The following are available online at https://www.mdpi.com/article/10.3390/ijms222413483/s1.

## Abbreviations

DIV	days in vitro
GluN1	NMDA receptor subunit 1
HGF	hepatocyte growth factor
NMDARs	N-methyl-D-Aspartate receptors
HGFR/MET	tyrosine-protein kinase Met or hepatocyte growth factor receptor (HGFR)
PLA	proximity ligation assay
sc-tPA	single-chain tissue-type Plasminogen Activator
tc-tPA	two-chains tissue-type Plasminogen Activator
