# Supplementary material for: Two-Chains Tissue Plasminogen Activator Unifies Met and NMDA Receptor Signalling to Control Neuronal Survival
Source: Int J Mol Sci. 2021 Dec 15;22(24):13483. doi: 10.3390/ijms222413483 (PMC8707453; doi:10.3390/ijms222413483)
Supplement: Supplementary file 1 [file ijms-22-13483-s001.zip › Hedou Supplementary Fig IJMS.pptx]

## Slide 1
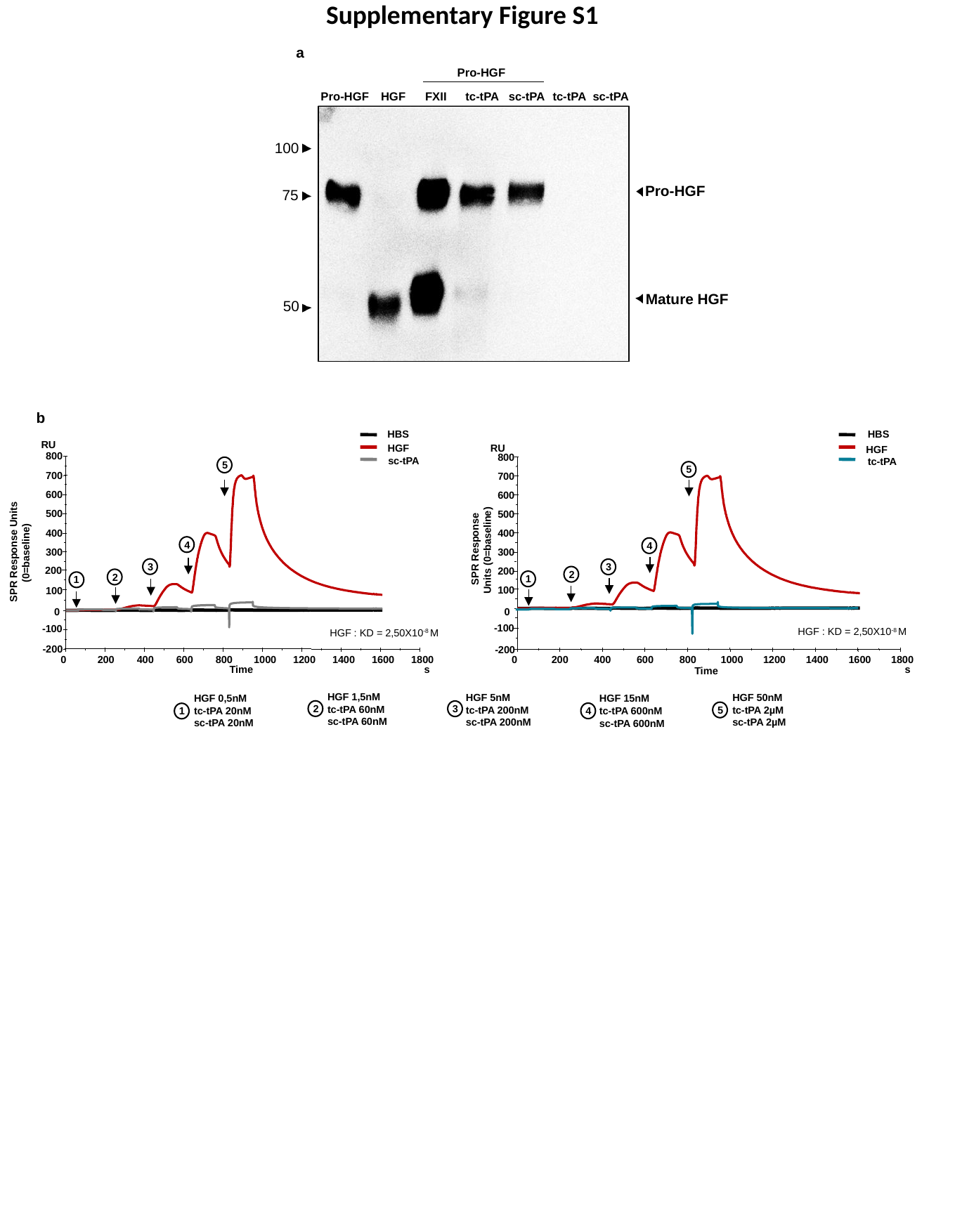

Supplementary Figure S1
a
Pro-HGF
Pro-HGF
HGF
FXII
tc-tPA
sc-tPA
tc-tPA
sc-tPA
100
Pro-HGF
75
Mature HGF
50
b
HBS
HGF
sc-tPA
HBS
HGF
tc-tPA
RU
RU
800
800
5
5
700
700
600
600
500
500
400
400
4
4
 SPR Response Units (0=baseline)
 SPR Response Units (0=baseline)
300
300
3
3
2
200
200
2
1
1
100
100
0
0
HGF : KD = 2,50X10-8 M
HGF : KD = 2,50X10-8 M
-100
-100
-200
-200
0
200
400
600
800
1000
1200
1400
1600
1800
0
200
400
600
800
1000
1200
1400
1600
1800
s
s
Time
Time
HGF 1,5nM
tc-tPA 60nM
sc-tPA 60nM
HGF 5nM
tc-tPA 200nM
sc-tPA 200nM
HGF 50nM
tc-tPA 2µM
sc-tPA 2µM
5
HGF 0,5nM
tc-tPA 20nM
sc-tPA 20nM
HGF 15nM
tc-tPA 600nM
sc-tPA 600nM
2
3
1
4

## Slide 2
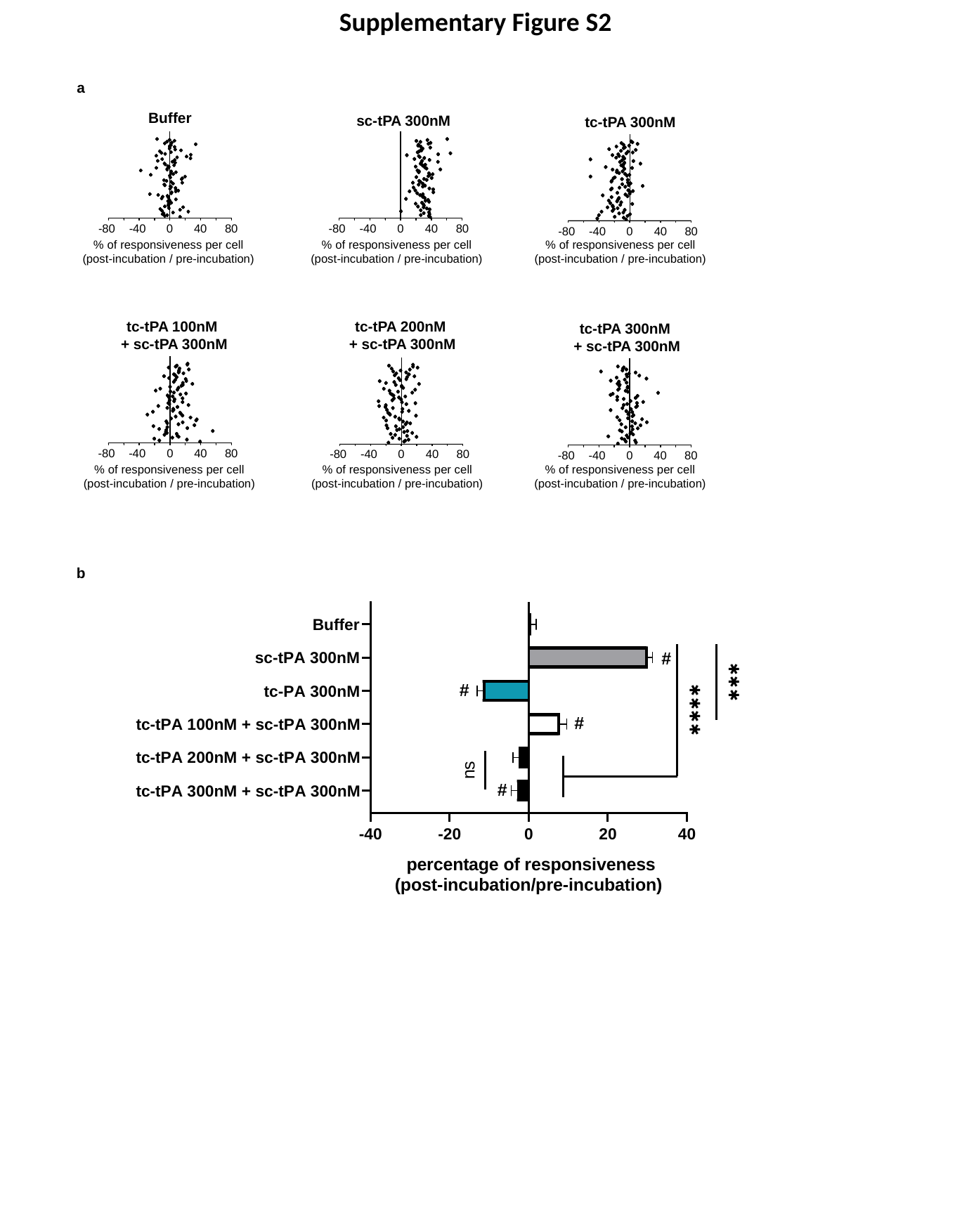

Supplementary Figure S2
a
Buffer
sc-tPA 300nM
tc-tPA 300nM
-80
-40
0
40
80
-80
-40
0
40
80
-80
-40
0
40
80
% of responsiveness per cell
(post-incubation / pre-incubation)
% of responsiveness per cell
(post-incubation / pre-incubation)
% of responsiveness per cell
(post-incubation / pre-incubation)
tc-tPA 100nM
+ sc-tPA 300nM
-80
-40
0
40
80
% of responsiveness per cell
(post-incubation / pre-incubation)
tc-tPA 200nM
+ sc-tPA 300nM
-80
-40
0
40
80
% of responsiveness per cell
(post-incubation / pre-incubation)
tc-tPA 300nM
+ sc-tPA 300nM
-80
-40
0
40
80
% of responsiveness per cell
(post-incubation / pre-incubation)
b
