# Supplementary material for: Two-Chains Tissue Plasminogen Activator Unifies Met and NMDA Receptor Signalling to Control Neuronal Survival
Source: Int J Mol Sci. 2021 Dec 15;22(24):13483. doi: 10.3390/ijms222413483 (PMC8707453; doi:10.3390/ijms222413483)
Supplement: Supplementary file 1 [file ijms-22-13483-s001.zip › Hedou Supplementary legends IJMS.pdf]

Supplementary figure S1: **sc-tPA and tc-tPA do not promote the activation of pro-HGF into active HGF**. (**a**), tc-tPA does not convert Pro-HGF in mature HGF. Pro-HGF (150 nM) was incubated at 37°C for 4 h without proteases (lane 1) or with 5,2 nM FXIIa (internal control), 150nM tc-tPA and 150nM sc-tPA. Immunodetection of αHGF after incubation. Representative images of three independent experiments are presented. (**b**) Resonance surface plasmon does not reveal of interaction of sc-tPA and tc-tPA on MET receptor. Five increasing doses (20nM, 60nM, 200nM, 600nM, 2µM) of sc-tPA and tc-tPA have been applied on fixed MET receptor and no SPR signal have been detected. HBS is the buffer used for the immobilization of MET receptor. Recombinant HGF has been used as control (0,5nM, 1,5nM, 5nM, 15nM, 50nM) showing an increase of SPR signal with an affinity (Kd) of 2,5x10-8M.

Supplementary figure S2: **sc-tPA and tc-tPA activate two different and opposite signaling pathways**. (**a,b**), Calcium video imaging performed on primary cultures of cortical neurons (12-13 DIV). **a**, After control NMDA stimulations used as baseline, neurons were incubated for 45min with buffers of tPAs (n = 90 cells), sc-tPA and tc-tPA (300nM; n = 83 cells and n = 92 cells respectively) or with sc-tPA (300nM) combined with tc-tPA (100nM, n = 82 cells; 200nM, n = 82 cells buffer; 300nM, n = 77 cells). Each dot represents one cell. **b**, Percentage of stimulation or inhibition after incubation were calculated for each individual cell and reported as the percentages of responsiveness for each group (n = 3 independent experiments, mean ± SEM) (+30.23% for sc-tPA, -11.65% for tc-tPA and +7.97% for sc-tPA + tc-tPA 100nM, -2.51% for sc-tPA + tc-tPA 200nM and -2.97% for sc-tPA + tc-tPA 300nM). #, indicates significant difference for the comparison of pre- and post-incubation responses by Wilcoxon signed-rank test ($p < 0.0001$). ****, indicates significantly different from the corresponding control by Kruskal-Wallis and Mann-Whitney tests ($p < 0.0001$).
